# Prevalence of asymptomatic malaria parasitaemia following mass testing and treatment in Pakro sub-district of Ghana

**DOI:** 10.1186/s12889-019-7986-4

**Published:** 2019-12-03

**Authors:** Ignatius Cheng Ndong, Daniel Okyere, Juliana Yartey Enos, Benedicta A. Mensah, Alexander Nyarko, Benjamin Abuaku, Alfred Amambua-Ngwa, Corinne Simone C. Merle, Kwadwo Ansah Koram, Collins Stephen Ahorlu

**Affiliations:** 1 Noguchi Memorial Institute for Medical Research, College of Health Sciences, University of Ghana, Legon, Ghana; 2 Department of Biochemistry, Faculty of Science, Catholic University of Cameroon, Bamenda, Cameroon; 3 Medical Research Council Unit, The Gambia at London School of Hygiene and Tropical Medicine, Serrekunda, Gambia; 4 Special Programme for Research & Training in Tropical Diseases (TDR), World Health Organization, Geneva, Switzerland

## Abstract

**Background:**

Global efforts to scale-up malaria control interventions are gaining steam. These include the use of Long-Lasting Insecticide Nets, Indoor Residual Spraying, Intermittent Preventive Treatment and Test, Treat and Track. Despite these, the drive for malaria elimination is far from being realistic in endemic communities in Africa. This is partly due to the fact that asymptomatic parasite carriage, not specifically targeted by most interventions, remains the bedrock that fuels transmission. This has led to mass testing, treatment and tracking (MTTT) as an alternative strategy to target asymptomatic individuals. We report the impact of MTTT on the prevalence of asymptomatic malaria parasitaemia over a one-year period in Ghana, hypothesizing that implementing MTTT could reduce the rate of asymptomatic parasitaemia.

**Methods:**

A population of about 5000 individuals in seven communities in the Pakro sub-district of Ghana participated in this study. A register was developed for each community following a census. MTTT engaged trained community-based health volunteers who conducted house-to-house testing using RDTs every 4 months and treated positive cases with Artemisinin–based Combination Therapy. Between interventions, community-based management of malaria was implemented for symptomatic cases.

**Results:**

MTTT Coverage was 98.8% in July 2017 and 79.3% in July 2018. Of those tested, asymptomatic infection with malaria parasites reduced from 36.3% (1795/4941) in July 2017 to 32.9% (1303/3966) in July 2018 (*p* = 0.001). Prevalence of asymptomatic parasitaemia among children under 15 years declined from 52.6% (1043/1984) in July 2017 to 47.5% (820/1728) in July 2018 (*p* = 0.002). Implementing MTTT significantly reduced asymptomatic parasitaemia by 24% from July 2017 to July 2018 after adjusting for age, ITN use and axillary temperature (OR = 0.76, CI = 0.67, 0.85 *p* ≤ 0.001).

**Conclusion:**

This study has demonstrated that implementing MTTT is feasible and could reduce the prevalence of asymptomatic malaria parasitaemia in children under 15 years of age. Furthermore, the use of community-based health volunteers could ensure high coverage at lower cost of implementation.

**Trial registration:**

NCT04167566, Date 14/11/2019. Retrospective registration.

## Background

Over the last two decades, tremendous progress has been made in the fight against malaria achieving significant reduction in the global prevalence by 18%, mortality and mobility in all age groups declined by 48%, while mortality in children 2–10 years declined by 33% [1, 2]. This has resulted from a step-up in control efforts toward its elimination [3]. Despite the recorded successes, malaria still remains a significant public health threat especially in endemic communities in Africa, where most of the morbidity and mortality occurs. Moreover, current evidence suggests a surge in prevalence and mortality between 2010 and 2016 in some parts of sub-Saharan Africa [4]. In order to change the present dynamics, there is a need to employ alternative intervention strategies such as mass testing, treatment and tracking (MTTT) [3, 5]. Malaria mass drug administration (MDA) is an effective and well-known strategy that has been found to drastically reduce prevalence, reverse mortality trends and led to elimination in specific areas [6–13]. MDA was used in the malaria eradication efforts at different points in history between 1950s – 1970s [6, 8, 14, 15].

MDA integrated with other approaches such as distribution of LLINs and strengthening community based health services and health workers, can accelerate malaria elimination ([16–19]. MDA as an approach has shown mixed results in the history of malaria elimination [8, 10, 20] and depends upon i) the targeted region’s epidemiology of malaria or transmission intensity; ii) geographical characteristics of the malaria endemic regions (islands where mobility of population is minimal have cleared malaria, such as Aneityum, Taiwan); iii) coverage of such an approach with effective community engagement [21, 22]; and the challenges could be resistance to antimalarials [19, 23–25] as well as lack of support from local authorities.

The factors that influenced the effectiveness of MDA remain broadly valid today with similar malaria epidemiology [15, 26, 27]. The renewed interest in malaria MDA has stimulated an interest in mass testing, treatment and tracking (MTTT) in order to limit unnecessary drug pressure and resistance development. MTTT may face similar challenges such as ineffective coverage and a lack of community ownership etc., requiring the following questions to be addressed; what level of community coverage can be achieved through MTTT? Can MTTT reduce malaria incidence? What will be the impact of MTTT on malaria-related hospital visits? We demonstrated in our earlier report that communities are willing to fully engage and take ownership of the MTTT interventions [28].

In the context of present day challenges, attempts have been made to formulate achievable malaria interventions such as passive testing, treatment and tracking (T3) of symptomatic cases at health facilities (standard of care), providing whole populations with long lasting insecticidal net (LLIN), intermittent preventive treatment in children (IPTc) and pregnant women (IPTp) and seasonal malaria chemoprevention (SMC) in sub-Sahara Africa [1, 29]. Today, these interventions mostly target passive cases detected or vulnerable groups such as pregnant women and children [30].

Though the above mentioned interventions have been shown to drastically reduce malaria prevalence, they remain insufficient in meeting the goal of reducing the prevalence of malaria to elimination levels. Malaria elimination is still far from being realistic in some endemic communities especially in Africa, under the present dispensation [24]. This is partly due to the fact that asymptomatic parasite carriage, not specifically targeted by most interventions, remains the bedrock that fuels transmission [3]. Malaria control strategies targeting asymptomatic malaria reservoir will have to deal with the social and cultural factors. For example, how local populations perceive the concept of sub-clinical/asymptomatic malaria [21, 28]. In addition, strategies targeting asymptomatic malaria would have to deal with intricate plural health care in such settings, where normal health seeking behaviour will be challenged. And such approach would have to weave through and build on an intricate health care seeking system through for example, collaborating with both formal and informal health care providers [31–34]. Given that cost is a major limitation for implementing MDA of malaria, MTTT is being proposed as an alternative strategy to target asymptomatic individuals, mitigate the effect of resistance development as well as limit unnecessary drug pressure on those who are not carrying the parasite [35, 36]. Attempts to implement mass treatment in endemic communities SMC are presently limited to children under 5 in the Sahelian region of Africa. SMC has been shown to reduce the burden of malaria in children below 5 years in Senegal, Mali and Burkina Faso and has further been proven to be affordable if extended to 10 year old children [29, 37–40]. While SMC has targeted the Sahelian region with marked seasonal transmission, strategies for reducing malaria parasitaemia towards elimination in other endemic transmission areas of Africa, which cover the broader population are needed.

Currently, symptomatic malaria still constitutes 38% of outpatient consultation in hospitals in Ghana, while asymptomatic malaria parasitaemia carriage is 25% in children under 15 and above 40% in school-age children [41–44]. Ghana adopted artesunate and amodiaquine as first line drugs for the treatment of malaria since 2006. It also adopted the T3 strategy in 2010, however, the return rate for malaria patients post-treatment is still below half [45]. Other drug interventions have included the implementation of IPTc complemented by community management of malaria in coastal communities, which resulted in a reduction of asymptomatic parasitaemia by 90% in children under 5 [46]. However, there are a number of unanswered questions, including: whether MTTT intervention could be scaled-up to the entire population since asymptomatic carriage is higher in adults who have developed partial immunity or premunition [47, 48]? What are the challenges to scaling up the intervention to the adult population? What effect MTTT would have on the prevalence of symptomatic parasitaemia in the short-term? In this article, we report findings from MTTT implementation across seven communities in the Pakro sub-district of Ghana over a period of 1 year, hypothesizing that implementing MTTT complemented by CBMm could reduce the prevalence of asymptomatic parasitaemia.

## Methods

### Study area

Pakro is one of five sub-districts in the Akwapim south district health directorate (DHD) in the Eastern region of Ghana [49]. The Akwapim south district lies within the semi-equatorial climatic region, and experiences two rainfall seasons in a year with an average rainfall of 125 cm to 200 cm. The first rainy season begins from May to June with the heaviest rainfall in June, whilst the second rainy season begins from September to October. According to the Ghana Statistical Service, the average household size in the Akwapim South district is 4.0 whilst the average number of households per house or compound is estimated to be 1.6 [50]. The Pakro sub-district has an estimated population of 7889 and is bounded to the east by Akwapim North district; to the north by Ayensuano district; and to the west by Nsawam Adoagyiri Municipality. The sub-district is made up of 22 communities, and has 4 health care facilities (1 Health Centre and 3 Community-based Health Planning Service (CHPS) compounds) [49]. The Pakro Health Centre is one of the 30 sentinel sites for monitoring malaria prevalence in the country coordinated by the Noguchi Memorial Institute for Medical Research. Malaria parasite positivity rate at the Pakro Health Centre was 45.7% in 2014 while anaemia among pregnant women at 36 weeks of gestation was 21% [49]. To undertake this study, 14 Community-Based Health Volunteers (CBHVs) were specifically recruited and trained for MTTT on the use of RDT test kits, treatment following the malaria treatment guidelines, and follow-up as well as reporting adverse events. CBHVs are community members without a health background.

### Selection of communities

Due to limited resources, seven communities were selected for this study; Abease Newsite, Fante Town, Zongo (Adjenase/Kweitey), Piem/odumsisi, Adesa, Sacchi/Tabankro and Odumtokro. These communities had relatively higher population densities. In consultation with the district health service, we considered a 5 km radius from the health facility and all 7 communities located within that zone were selected. Additionally, they are served by two public health facilities – the Pakro sub-district Health Centre and the Zongo CHPS compound. Patients from all 7 communities visit the health facilities to seek health care. Staff from the Health facilities undertake outreach services to the different communities.

### Study participants

**Fig. 1:**
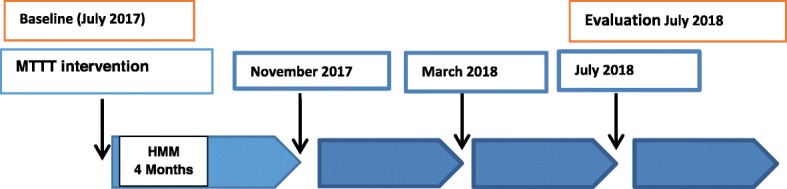
Implementation scheme of MTTT interventions in Pakro. MTTT interventions were conducted every 4 months. Between interventions, the CBHVs conducted community-based management of malaria. The time *indicates the intervention periods. This report compares the parasitaemia prevalence at baseline to evaluation*

The entire population of about 5000 from the seven selected communities in Pakro sub-district was enrolled in the study. This population size was obtained through the household census. Community engagement activities to sensitize the chiefs and the general population was conducted at the beginning of the study through meetings and durbars [41, 51]. All households were numbered, and community registers developed to ensure tracking of the participants. Each household was given a unique identification code. Each individual within the household was assigned a code that links them to a particular household and community. After obtaining parental informed consent, the children were enrolled but individual assent and consent was obtained from the adolescents and adults. (Fig. 1).

### Inclusion criteria

All community members were included in the study population. Willingness to participate was evident upon completion and signing of a consent form by the individual, parent or guardian in the case of children.

### Exclusion criteria

If an individual had a life-threatening illness (excluding malaria) (s) he was excluded. However, all individuals including those with clinical malaria signs who were present during surveys were tested and when confirmed to carry malaria parasites were treated.

### MTTT of the population

In this study we considered asymptomatic parasitaemia as a positive RDT test with no history of fever prior to survey, axillary temperature of less than 37.5 °C and no other signs and symptoms of malaria such as headache, vomiting, abdominal pain, nausea or diarrhoea. The entire population of the selected communities was screened for the presence of malaria parasites using the Ag P.f RDT (SD Bioline, Standard Diagnostics, Republic of Korea) which detects histidinie-rich proteins II antigens (HRP-2 Ag) specific to *P. falciparum* in human blood. The RDTs were obtained from the National Malaria Control Programme. All participants confirmed to be carrying malaria parasites were treated using an ACT following the National Malaria Treatment Guidelines [14]. To ensure that participants adhered to treatment and to document adverse events, the first dose was directly observed and participants followed-up at home to confirm treatment adherence on days 1, 2, 3 and 7. Each participant was observed for 5 minutes after treatment administration to ensure that they retain the drug. For children who vomited within 5 minutes of taking ACT, the treatment was re-administered as it was assumed that the drug had not been adequately absorbed.

The ACTs used in this study were also obtained from the National Malaria Control Programme. The drug regimen was changed at any given time depending on what the NMCP was supplying across the country. For instance, in July 2017, March 2018 and July 2018, we used artesunate-amodiaquine (AA) while in November 2017 we used artemether lumefanthrine (AL).

### Timely treatment of suspected febrile malaria cases in the community

To facilitate MTTT, two CBHVs from each community were recruited, trained and provided with the protocol, RDTs and ACTs. Between one MTTT intervention and the next, all children and adults reporting signs and symptoms of malaria were tested by CBHVs using RDTs. When confirmed to be carrying the malaria parasite, the CBHVs treated the participant promptly using ACTs following the malaria management protocols provided. The research team conducted monthly monitoring visits to interact with CBHVs involved in community-based management to ensure that the protocol was being respected and to replenish their stocks.

### Data collection

To facilitate tracking and improve coverage, a community register was developed. Communities were divided into neighbourhoods and CBHVs were assigned a specific catchment area where they are well known. This allowed the CBHVs to schedule appointments with a particular household head before visiting to collect data. This enabled the CBHVs to visit when most of the household members were at home. In some instances, the CBHVs had to visit the houses more than once to be able to attend to all participants.

Following consent, axillary temperature of participant was recorded using a digital thermometer and blood was drawn from a finger prick for malaria testing. At baseline, all participants were tested using RDTs (prevalence survey) before treatment with ACT (intervention), when positive. The data resulting from this work were stored at the Noguchi Memorial Institute for Medical Research. Hospital data was also collected during the intervention months.

### Data management and analysis

Data was analysed using SPSS (IBM SPSS Statistics 20, United States). The unit of enrolment was the household. Malaria prevalence was reported as proportion of participants confirmed during screening to be carrying the malaria parasite, and they were stratified by demographic variables such as age, sex and community. To determine whether treating asymptomatic parasitaemia had an effect on the incidence of symptomatic malaria during OPD attendance, hospital data were compared to intervention data. A Chi square statistic was used to compare prevalence of parasitaemia across age groups, gender and communities at the 95% confidence level (*p* = 0.05). The *p*-values represent changes between temporal time points. Also, a regression analysis was conducted to test the effect of factors such as timeline, age, temperature and use of LLINs on the MTTT intervention.

## Results

### Coverage

Four MTTT interventions were conducted in July 2017, November 2017, March 2018 and July 2018. Of the 5000 participants initially targeted across communities for each intervention, coverage ranged from 77.8% (3891/5000) to 98.8% (4941/5000) (Table 1). A total of 2669 participants received all four MTTTs (53%) and the median number of MTTTs participants received was 3. The proportions represent those who were effectively reached and tested. Females made up 50.4 and 52.4% of participants in July 2017 and July 2018, respectively (Table 1). The fluctuation in coverage was largely due to movement of people in and out of the intervention area, and the proportion represents those who were effectively reached. If a household was visited three times and the participant could not be contacted, (s) he was considered not available for the survey/intervention or reported to have moved.

**Table 1:** Parasite carriage across different communities at different time points in the Pakro sub-district

Characteristics	Prevalence of asymptomatic parasitaemia among community members N (%)			
	July 2017 Survey n/N (%)	November 2017 Survey n/N (%)	March 2018 Survey n/N (%)	July 2018 Survey n/N (%)
Community				
Abease	282/888 (31.8)	124/712 (17.4)	77/709 (10.9)	161/721 (22.3)
Adesa	158/406 (38.9)	85/323 (26.3)	62/351 (17.7)	121/342 (35.4)
Adjanase	514/1342 (38.3)	191/957 (20.0)	283/1070 (26.5)	387/1037 (37.3)
Fante Town	543/1530 (35.5)	306/1238 (24.7)	347/1284 (27.0)	392/1236 (31.7)
Odumtokro	101/245 (41.2)	42/213 (19.7)	43/190 (22.6)	63/180 (35.0)
Piem	80/220 (36.4)	35/183 (19.3)	50/157 (31.9)	57/160 (35.6)
Sachi/Tabankro	117/310 (37.7)	75/265 (28.3)	75/277 (27.1)	122/290 (42.1)
Total	1795/4941 (36.3)	858/3891 (22.1)	937/4038 (23.2)	1303/3966 (32.9)
Coverage	4941/5000(98.8)	3891/5000(77.8)	4038/5000(80.8)	3966/5000 (79.3)

### Asymptomatic parasitaemia prevalence

Considering the different time points across the intervention period, asymptomatic malaria parasite carriage was found to be 36.3% in July 2017, 22.1% in November 2017, 23.2% in March 2018 and 32.9% in July 2018. These observations reflect the seasonality of malaria in the district and across the country.

Comparative analysis between the two time points, July 2017 to July 2018 revealed a decline in asymptomatic parasite carriage from 36.3% (1795/4941) to 32.9% (1303/3966) (*p* = 0.001) (Tables 1 and 2)). The highest level of community-specific prevalence of asymptomatic parasitaemia per time point were 41.2, 28.3, 31.9 and 42.1% for July 2017, November 2017, March 2018 and July 2018 respectively (Table 1). Parasite carriage significantly declined in both Abease and Fante Town between July 2017 and July 2018 (*p* < 0.0001 and *p* = 0.037 respectively), but, the decline in the other communities was not statistically significant. An increase in parasite carriage was observed for Sachi/Tabankro from July 2017 to July 2018. However, this was not significant, (Table 2).

**Table 2:** Univariate analysis of effect of MTTT interventions on prevalence of asymptomatic malaria parasitaemia over the time points July 2017 and 2018

Characteristics	July 2017 Survey	July 2018 Survey	χ^2^ value	*P* value
Community	n/N (%)	n/N (%)		
Abease	282/888 (31.8)	161/721 (22.3)	17.1	< 0.001**
Adesa	158/406 (38.9)	121/342 (35.4)	1	0.319
Adjanase	514/1342 (38.3)	387/1037 (37.3)	0.2	0.624
Fante Town	543/1530 (35.5)	392/1236 (31.7)	4.4	0.037**
Odomtokro	101/245 (41.2)	63/180 (35.0)	1.7	0.193
Piem	80/220 (36.4)	57/160 (35.6)	0	0.882
Sachi/Tabankro	117/310 (37.7)	122/290 (42.1)	1.17	0.279
All Communities	1795 (36.3)	1303 (32.9)	11.71	0.001**
Age_group (years)	n/N (%)	n/N (%)		
0–11 months	14/56 (25.0)	22/57 (38.6)	2.4	0.121
1–4 years	270/541 (49.9)	216/491 (44.0)	3.6	0.057
5–14 years	759/1387 (54.7)	582/1180 (49.3)	7.5	0.006**
15–45 years	5,852,092 (28.0)	360/1523 (23.6)	8.5	0.003**
46–65 years	129/640 (20.2)	97/544 (17.8)	1	0.310
> 65 years	38/225 (16.9)	26/171 (15.2)	0.2	0.352
Total # of < 15	1043/1984 (52.6)	820/1728 (47.5)	9.7	0.002**
Total # of ≥ 15	752/2957 (25.4)	483/2238 (21.6)	10.4	0.001**
Mean axillary Temperature	36.0	36.2		< 0.001**
Use ITN				
No	1595/4574 (34.9)	380/1166 (32.5)	0.0012	< 0.001
Yes	200/367 (54.5)	923/2800 (33.0)		

All percentages represent proportions of parasite carriage compared to the population tested. The comparison is made only between July 2017 and July 2018 which depicts the same season

** Significance level at α =0.05

The highest decline in parasitaemia carriage, was observed in Abease while the lowest decline was observed in Adesa. It is not clear what accounted for the heterogeneity as there were no other interventions in the area at the time of this study. Asymptomatic parasitaemia prevalence from July 2017 and July 2018 significantly decreased in both children and adults (*p* = 0.002 for < 15 children and *p* = 0.001 for ≥15 years) (Table 2). As per age group, asymptomatic parasitaemia prevalence significantly declined in the age groups 5–14 and 15–45 years, but the decline was marginally significant in the age group 1–4. This decline in parasitaemia was not significant in the age groups 46–65 and >  65 years. An increase in parasitaemia prevalence was observed among the < 1 year old age group between July 2017 and 2018. But this was not significant.

A logistic regression was performed to determine the predictors of malaria parasitaemia and the magnitude of the effect of the intervention on the outcome of interest, which is malaria parasitaemia. Age, use of ITN and axillary temperature were predictors of malaria parasitaemia at the univariate level (α =0.05) (Table 3) and were included as confounders in predicting the magnitude of effect of the intervention on malaria parasitaemia prevalence. The intervention reduced parasitaemia by 24% a year, after the mass treatment in July 2018, following adjusting for confounders (OR = 0.76, CI = 0.67, 0.85 *p* value ≤0.001) and by 9.4% a year, unadjusted. A unit increase in age reduces malaria prevalence by 3% after adjusting for confounders (OR = 0.97, CI = 0.97, 0.97, *p* value≤0.001). The use of ITN was not a statistically significant predictor of malaria parasitaemia prevalence after adjusting for confounders (OR = 1.11, CI = 0.98, 1.26, *p* value ≤0.001). The implementation of MTTT reduced symptomatic parasitaemia by 9% from July 2017 to July 2018 after adjusting for age, though not statistically significant (OR = 0.91, CI = 0.67, 1.38 *p* value = 0.672) (Table 4).

**Table 3:** Logistic regression for asymptomatic parasitaemia

Characteristics	Unadjusted OR	*P* value	Adjusted OR	*P* value
Timeline				
July 2017 survey	Ref	0.001	Ref	< 0.001
July 2018 survey	0.86 (0.78, 0.94)		0.76 (0.67, 0.85)	
Age (years)	0.97 (0.97, 0.97)	< 0.001	0.97 (0.97, 0.97)	< 0.001
Mean axillary Temperature	1.19 (1.14, 1.25)	< 0.001	1.19 (1.12, 1.26)	< 0.001
ITN Use				
No	Ref	0.034		0.086
Yes	0.92 (0.87, 0.99)		1.11 (0.98, 1.26)

**Table 4:** Logistic regression for symptomatic parasitaemia

Characteristics	Unadjusted OR (CI)	P value	Adjusted OR (CI)	*P* value
Timeline				
July 2017 survey	Ref	0.381	Ref	0.672
July 2018 survey	0.83 (0.57, 1.24)		0.91 (0.67, 1.38)	
Age_group (years)				
0–11 months	Ref	< 0.001	Ref	< 0.001
1–4 years	1.72 (1.41, 2.11)	< 0.001	0.67 (0.64, 6.91)	0.53
5–14 years	3.31 (2.70, 4.08)	< 0.001	1.62 (0.44, 5.92)	0.469
15–45 years	0.72 (0.59, 0.89)	0.002	0.54 (0.15, 1.86)	0.328
46–65 years	0.46 (0.38, 0.58)	< 0.001	0.31 (0.08, 1.16)	0.082
> 65 years	0.49 (0.40, 0.61)	< 0.001	0.34 (0.09, 1.23)	0.111

### Asymptomatic versus symptomatic malaria parasite carriage

Records show that in July 2017, the Pakro Health Centre was visited by 4.4% (219/4941) of the study population who became febrile. Of these, 1.9% (96/4941) were confirmed to be positive for malaria. This means that 43.8% (96/219) of all those presenting with fever were positive for symptomatic parasitaemia carriage in July 2017 and were treated. In July 2018, 5% (197/3966) of the population that became febrile, visited the Health Centre and 2% (78/3966) of the population were confirmed to be positive for malaria. Prevalence of symptomatic parasitaemia among those presenting with fever was 39.6% (78/197) representing a 4.2% reduction in symptomatic parasitaemia between July 2017 and July 2018 (Table 5). Implementing MTTT interventions significantly reduced asymptomatic parasite carriage by 24% (OR = 0.76, CI = 0.67, 0.85 *p* = ≤0.001) after adjusting for age, use of ITNs and axillary temperature, compared to a non-significant reduction of 9% (OR = 0.91, CI = 0.67, 1.38, *p* = 0.672) in confirmed symptomatic malaria cases who attended the health facility over the same period (Table 5). This demonstrates that implementing MTTT interventions could reduce the asymptomatic malaria prevalence, which may not necessarily be reflected to the same extent in the outpatient consultations within the first year.

**Table 5:** Comparative analysis of asymptomatic and symptomatic prevalence in the Pakro sub-district

	MTTT Data (Asymptomatic parasitaemia)			Hospital Data for clinical malaria (symptomatic parasitaemia)		
	RDT +	Total tested	%	RDT +	Total OPD attendance	%
Jul-17	1795	4941	36.3	96	219	43.8
Jul-18	1303	3966	32.9	78	197	39.6

Confirmed parasitaemia was found to have decreased across all the age groups except the 0–11 months and 15–45 years age group where the proportion increased. However, neither the observed increase nor decrease in parasitaemia was significant in all age groups (Table 6).

**Table 6:** Confirmed symptomatic parasitaemia across all age groups N (%)

Age group	July 2017 Survey	July 2018 Survey	χ^2^ value	*P* Value
0–11 months	2/5 (40.0)	4/6 (66.7)	0.7822	0.567
1–4 years	28/60 (46.7)	16/38 (42.1)	0.1957	0.658
5–14 years	24/33 (72.7)	17/29 (58.6)	1.3713	0.242
15–45 years	26/73 (35.6)	23/51 (45.1)	1.1293	0.288
46–65 years	6/18 (33.3)	11/45 (24.4)	0.5156	0.473
> 65 years	10/30 (33.3)	7/28 (25.0)	0.4854	0.486
Total # of < 15	54/98 (55.1)	37/73 (50.7)	0.3279	0.567
Total # of ≥15	42/121 (34.7)	41/124 (33.1)	0.0741	0.785

## Discussion

### Coverage

Mass testing, treatment and tracking of the entire population in an endemic area such as Pakro is suggested to contribute to a decline in asymptomatic malaria parasite carriage. Population coverage of more than 75% could be achieved during MTTT interventions by using CBHVs [6]. Though some level of population movement in and out of the study communities was observed, the communities are more or less established with a relatively low degree of migration. This is promising for malaria elimination efforts through MTTT in endemic areas [11]. However, there is a need to validate these findings in a larger population by scaling up the intervention.

### Effect of MTTT on asymptomatic parasitaemia

The reasons for variation in parasite carriage and impact of MTTT across communities is not clear. Malaria prevalence across the communities was heterogeneous and we suspect that differences in environmental factors which play a role in malaria transmission in the locality could be contributing to the heterogeneity in outcome. Abease and Odomtokro are higher in altitude while the rest of the communities are low lying, forming part of the flood plain of the Densu River, which could be affecting the continuous transmission in the area. Our results suggest that if MTTT is abandoned without ensuring that the parasite is effectively cleared from the community, transmission could easily be re-established to previous levels within a short time [6]. This is because the drop in asymptomatic parasitaemia of 3.4% between the two time points under consideration was relatively low.

As expected, there was decline in asymptomatic malaria for both children under-15 and those above 15 years. Participants aged 15 years and above recorded the highest asymptomatic parasite carriage rate and incidentally is the age group that does not usually experience febrile illness. Therefore, reducing the malaria parasite carriage in this age group could potentially propel a decline in transmission, which could lead to a decline in the burden of febrile malaria in the children under 15, especially among the under-5 children who have not yet developed partial immunity to the malaria parasite [3, 31, 32, 41, 52]. There were no significant differences in the symptomatic burden in both children < 15 and those above 15 years between July 2017 and July 2018. There is need for more data to inform meaningful conclusions from these observations.

### Asymptomatic versus symptomatic parasite carriage

Registering more cases of asymptomatic parasite carriage through MTTT, compared to symptomatic cases through the standard of care (T3) at the health facilities is of public health relevance. This observation suggests that malaria control and intervention strategies should consider asymptomatic parasitaemia as a public health concern and a major hindrance to malaria elimination efforts. Considering that 94% of the asymptomatic malaria carriers in the study area did not become febrile should probably suggest that malaria elimination programmes in endemic communities such as Ghana, may require a change in strategy from targeting symptomatic to asymptomatic parasite clearance. While clearing of asymptomatic parasitaemia can lead to a decline in transmission over time [3] and consequently reflect in a reduction in malaria-related hospital attendance, the effect may or may not be very visible in the first years of MTTT implementation. This is more-so if malaria-related visits is considered as a lone indicator rather than all-cause hospital visits [23, 53]. This finding is in sharp contrast to the report by Halliday et al., [54] who reported the absence of an impact on health following the intermittent preventive treatment of school age children in Kenya. It seems a public health dilemma, that in Pakro less than 10% of the malaria parasite carriers actually visit health facilities for clinical management. Despite this observation, the health facility complained that during the MTTT interventions, there was a drastic drop in the number of febrile cases attending the facility. This was further confirmed by the perceptions of the community members during focus group discussions, when they stated that their level of hospital attendance had reduced during that period [28]. It has been reported that a decline in malaria episodes in Kenya was accompanied by a concurrent decline in bacterial diseases [55]. The observations in Kenya could potentially explain our observations in Pakro sub-District, though the rate of bacterial causes of hospitalizations was not determined in this study.

Also, since malaria constitutes the bulk of OPD attendance, eliminating malaria in endemic communities could pose other public health concerns which need to be anticipated as other programmes seem to depend on revenue generated from malaria visits to run the health facilities [52]. As reported in our earlier publication, implementing MTTT led to a decrease in the internally generated revenue of the Pakro Health Centre [28]. Rather than being a challenge, this is an important indicator of the outcome of the study which could help inform reforms in the health sector in the wake of efforts to eliminate malaria in endemic and resource limited settings. This could mean that under funded programmes in the health sector, which depend on revenue generated from malaria management in health facilities will need to reconsider their options.

### Limitations

Some of the limitations of this study include the following: i) The duration of the study was rather short, enabling minimal data collection which could not adequately explain all the observation made in this study. Much more extensive data need to be collected in future studies to enable more robust observations and conclusions. ii) The effect of community-based management of malaria by the CBHVs on the outcome of the study was not assessed and quantified. This could have thrown more light on the impact of MTTT in the communities studied. iii) The parasite status of participants who moved in and out of the study communities between interventions was not assessed to determine whether they returned with new infections iv) We used only RDTs for testing malaria parasitaemia. This means we might have missed participants with low density parasitaemia which could not be detected by the RDTs. These observations could have impacted the results of the study. v) Also, we did not have a control arm for this study. This could have provided further insight to the outcome of this study. This component will be included in future studies.

## Conclusion

The findings of this study demonstrate that MTTT contributed to a decline in asymptomatic malaria parasite carriage among the population of the Pakro sub-district of Ghana. It is feasible to attain more than 75% coverage of MTTT complemented by community-based management of malaria in Pakro and similar endemic areas. The study also revealed that most parasite carriers in endemic communities do not become febrile, thus, more people can be cleared of the parasite through MTTT than the current facility-based consultations or standard of care (T3) that focuses on symptomatic patients. Furthermore, the results suggest that in the early stages of implementing MTTT, reduction in asymptomatic parasite carriage in the community may or may not immediately be reflected in malaria-related attendance at health facilities in the short term.

## Data Availability

The data analysed is available in the Department of Epidemiology, Noguchi Memorial Institute for Medical Research, University of Ghana and can be made available by the corresponding author upon reasonable request. The datasets used in the study are available from the corresponding author on reasonable request.

## Abbreviations

AA: Artesunate Amodiaquine
ACT: Artemisinin-based Combination Therapy
AL: Artemether Lumefantrine
CBHV: Community-Based Health Volunteer
CHPS: Community-based Health Planning and Services
ERC: Ethics Review Committee
FGD: Focus Group Discussion
GHS: Ghana Health Service
HMM: Home-based Management of Malaria
IPTc: Intermittent Preventive Treatment in children
IPTp: Intermittent Preventive Treatment in pregnant women
IRB: Institutional Review Board
IRS: Indoor Residual Spraying
LLIN: Long Lasting Insecticidal Nets
MTTT: Malaria Testing, Treatment and Tracking
SMC: Seasonal malaria chemoprevention
T3: Test Treat Track
WHO: World Health Organisation
